# Supplementary material for: Predictors of development of cardiac and digestive disorders among patients with indeterminate chronic Chagas Disease
Source: PLoS Negl Trop Dis. 2021 Aug 13;15(8):e0009680. doi: 10.1371/journal.pntd.0009680 (PMC8386853; doi:10.1371/journal.pntd.0009680)
Supplement: Attachment — Submitted filename: chagas evolution review comorbidities.docx [file pntd.0009680.s003.docx]

The authors analyze data collected at their institution on 379 persons with the indeterminate form of Chagas disease followed during a 20-year period. Their study population is of special interest because of the high proportion of subjects with cardiovascular comorbidities and risk factors for cardiac disease (hypertension, myocardial infarction, hyperlipidemia, diabetes); in addition, the median age of subjects is substantially greater than that of earlier studies of younger cohorts of persons with the indeterminate form. As such, the authors' study population presents an opportunity to shed light on the evolution of Chagas heart disease among older persons and persons with comorbidities, who now represent a major proportion of persons infected with T. cruzi. However, as analyzed and presented, the study offers few insights into these important groups of persons with chronic infection, and conclusions about the impact of benznidazole on evolution of heart disease may be misleading. In contrast, the data and analysis on gastrointestinal disease seem more robust in part because few persons in the population had underlying gastrointestinal disease or neurological disease that might predispose to chronic Chagas GI disease.

The authors do not define what is meant by the indeterminate form of Chagas disease. The traditional definition includes a normal electrocardiogram (ECG); however, a high percentage of participants had heart disease (defined in later tables as myocardial infarction) or hypertension, and I suspect that some (if not many of these) had some type of abnormality on ECG. Roughly 73% of persons who developed the cardiac form of Chagas disease had hypertension on initial examination and 32% had heart disease (“myocardial infarction”); the corresponding number of persons who did not progress were 50.5% (97/192) and 8% (16/192); respectively.

The cardiac endpoints are defined as “abnormalities in those tests that could not be attributed to other causes” (table 2); RBBB is suggestive of Chagas disease, but neither RBBB or any of the listed abnormalities are completely specific for Chagas disease. LBBB (accounting for >25% of the abnormalities in table 2) occasionally can be attributed to Chagas disease, but is much more likely in this population to be a consequence of hypertension. The same can be true of LV dysfunction and other abnormalities in table 2.

The methods section states that all studies were repeated yearly—if so, this would be an unusually high rate of follow-up for a large population followed over a 20 year period. However, some of the abnormalities listed in table 2, such as atrial fibrillation, dilated cardiomyopathy right, left bundle branch block and others would never develop in one year’s time in an infected person with a normal ECG and echocardiogram.

In population-based studies of Chagas cardiomyopathy, the usual evolution is for early development of RBBB and then progression to more advanced disease; persons in the indeterminate stage who have not developed evidence of heart disease tend to remain in the indeterminate stage for decades. Many of the patients in the present study probably had been infected for many years or decades at time of entry, based on their ages and the fact that transmission of T cruzi had been interrupted in much of Brazil (and certainly in Sao Paulo state) prior to their enrollment. Thus, development of cardiac abnormalities may have been due to processes other than Chagas disease.

If I’m reading the data in the supplementary file correctly, of the 56 persons with underlying heart disease, only 5 received benznidazole; the percentage of persons without underlying heart disease who received benznidazole is >2x greater; this suggests that presence or absence of heart disease may have influenced the decision to administer benznidazole. Given the absence of criteria that determined which patients received benznidazole, conclusions cannot be drawn about its efficacy in preventing the development of Chagas heart disease.

In summary, the authors have compiled a potentially important data set that could shed light on the evolution and prognostic factors for Chagas heart disease in persons with comorbidities, cardiac risk factors, and older age. The authors should consider reanalysis of the data comparing outcomes in persons with pre-existing conditions to those without and controlling for age. Given the small numbers of persons who received benznidazole, it may not be possible to draw conclusions about the effect of benznidazole in different groups. As the authors acknowledge, randomized controlled studies are essential for evaluating the impact of benznidazole.

Specific minor comments:

Page 2, lines 6-7. The authors might point out that the study was carried out at a time in which most transmission in Southeastern Brazil had been interrupted.

Page 3. Migration is unlikely to lead to major spread of infection within Europe in North America by transfusion or other routes

Page 4.

Line 3. Many persons with Trypanosoma cruzi infection live outside of South America; would not say that almost all of them live in South America.

Line 5. Oral and parenteral transmission has been known for a long time; would not say that they were reported more recently.

Line 7-8. Extensive migration has already led to large numbers of infected persons in Europe North America.

Page 5. It would be important to clearly define what the authors mean by indeterminant form of Chagas disease. As mentioned above, many of the subjects had a history of underlying heart disease and may not fit the strict definition of indeterminant form.

Table 1. "Heart disease" later is defined as myocardial infarction.

Table 3. Would define what is meant by “colon abnormalities”

Figure 1. The curve extends to greater than 400 months although the study lasted only 240 months. Not clear which meant by cumulative survival

Page 15

Line 2-4. It was not just urbanization and improvement of housing but also the national control program that reduced transmission of the parasite.

Line 6. If there are 7 million infected persons, greater than 1-2 million would have the indeterminate form.

Line 11-12. Rate of progression from indeterminate form to cardiac form varies by age and is not 5% across all age groups.
