# Supplementary material for: Predictors of development of cardiac and digestive disorders among patients with indeterminate chronic Chagas Disease
Source: PLoS Negl Trop Dis. 2021 Aug 13;15(8):e0009680. doi: 10.1371/journal.pntd.0009680 (PMC8386853; doi:10.1371/journal.pntd.0009680)
Supplement: Attachment — Submitted filename: RESPONSE TO REVIEWERS.docx [file pntd.0009680.s004.docx]

**RESPONSE TO REVIEWERS**

To the Editor and Reviewers –

The authors thank you for carefully reading our manuscript and for your useful comments and recommendations. We address each topic separately bellow. Changes in the manuscript are presented in red types.

*Reviewer #1: There is a need for important clarifications on study design and methodology. Though the design is described as non-concurrent design, it is not totally clear if the study was prospective or retrospective. It would be important to present and review the protocol of the study.*

*An important consideration for this type of study is the standardisation of assessments performed over time and the treatment of missing data. No detailed information is provided on any of the assessments for disease progression (echo, electrocardiographic readings, and evaluation of digestive manifestations) - it is unclear which parameters were to be tracked were defined a priori, if different assessors were involved, if there was independent review of information, what were the data collection methods, etc. Also there is no information on Benznidazole treatment, dose, compliance and assessment of response.*

**Authors’ response:** Thank you for your comments. The term “non-concurrent” refers to what is usually described as a “retrospective cohort”. Authors such as Szklo & Nieto (Epidemiology – beyond the basics. 4^th^ Ed. Burlinton, MA: Jones & Bartlett, 2019) state that no cohort is indeed retrospective, since the rationale departs from exposure to outcome (and not the opposite way, like in case-control studies). Therefore, they recommend the term “non-concurrent” rather than “retrospective”, but we did include the last term to make sure that readers understand the method. We also changed the methods section in order to clarify the aspects pointed by the reviewer.

*Reviewer #2: Objectives*

*- The study has the objective to determine predictors for the development of cardiac and digestive disorders among patients with chronic and indeterminate Chagas' Disease. The study's purpose is outstanding. Such determinants can enlighten clinicians on the particular variables that are most relevant for monitoring patients, and that can ascertain the conduct of the medical team. The findings would also have the potential to establish a new hypothesis to incentivize new research avenues for the study of Chagas' Disease.*

**Response**: We thank you for your kind comments.

*- The central idea of the study's aim is to determine predictors for the development of cardiac and digestive disorders. However, on page 6, lines 1-2, the authors casually mention in a short sentence that they will also investigate the use of Benznidazole. Nevertheless, the authors have a great focus on the use of the drug in the "Results" section, and the majority of the "Discussion" section is used to address the use of this anti-parasitic drug. Therefore, there is a discrepancy between the study's goals and what the study ended up focusing on. The findings on this drug are relevant and should be described in the study. Still, the author should make it clear to the reader that the study has more than one aim: the first being determining predictors of cardiac and digestive disorders in Chagas' Disease patients; the second being if there is an association between Benznidazole and the outcomes of interest, and if this may suggest a p*rotective eff*ect. The same clarity should be provided in the "Introduction" section and the title of the manuscript.*

**Response:** We changed the title and introduction accordingly.

Study design and population

*- No mention is made in the manuscript to describe if the sample size sufficient ensures adequate power to address the hypothesis being tested. However, the study uses a convenient sample of all the patients with a Chagas' Disease diagnosis who were admitted while presenting the disease's indeterminate chronic form (ICF), and the study does yield statistical significance.*

*- The study is defined as a "non-concurrent cohort." Despite its description of outcomes, as the development of cardiac and digestive disorders, no clear distinction is made in the "Methods" section about what constitutes the "exposure" as it is inherent expected in a cohort study. The authors do not mention the "prospective" nature of the study, but the data collection is prospective and obtained from patients who enrolled between 1998-2018.*

**Response:** The term “non-concurrent” is particularly appropriate to our study, because patients were followed prospectively in our outpatient service, with a standardized protocol, but study data were collected retrospectively from medical charts. That is in accordance with Szklo & Nieto’s statements that “every cohort is prospective”.

*- On Page 5, lines 9-10, the authors describe the research subjects as participants who were admitted with the indeterminate chronic form of Chagas’ disease between the years 1998-2018, but that data was collected until 2019. To reduce the chances for confusion, I advise the authors to make it clearer for the readers that 1998-2018 was the window for enrollment of the recruited patients, but that data was obtained from beyond the enrollment window, and therefore, 2019 is also included.*

**Response:** This aspect was clarified in the manuscript.

*- Inclusion criteria were defined as "patients with CD who were admitted while presenting the ICF in the period from year 1998 through 2018" (page 5, lines 8-10). Exclusion criteria were defined as "Those who presented abnormalities upon admission."*

**Response:** As inherent to the cohort study, no subject presents the outcome of interest when included in the study. That is the reason for our exclusion criteria.

Statistical analyses

*- Statistical analyses utilized univariate and multivariable Cox regression models, which could be considered appropriate to assess associations between particular variables and the outcomes of interest. As a suggestion, the author could have also used data mining to study predictive models of associations among the variables.*

**Response:** Data mining is an exciting venue for modeling. Unfortunately, following that recommendation would lead to a complete re-design of our study, which is impossible in the present.

*- The authors were unclear as to which variables were considered for each of the multivariable models. The authors mentioned that variables were included gradually in multivariable models using a stepwise forward strategy. However, no additional documents were provided with the statistical analyses to clarify the variables used for each measurement.*

*- The authors should describe in detail in the "Methods" section, which variables were included in each of their multivariable models. This practice refines the reader's understanding of which variables have an association with the outcomes of interest in others' presence. The authors should also provide additional documentation from their statistical analysis performed using SPSS 20.*

*- There are no mentions of possible bias, confounders or effect modifies that could have interfered with the analyses.*

**Response:** There are several strategies for variable selections for multivariable analysis, which are discussed in classic papers such as Greenland S. Modeling and variable selection in epidemiologic analysis. Am J Public Health. 1989 Mar;79(3):340-9. We used what Greenland terms “conventional stepwise approach” with forward strategy. Quoting his paper: “Among all candidate variables not in the current model, find the one that would have the most significant coefficient if entered in the model; if its coefficient would be "statistically significant" (e.g., p < 0.05), enter it into the model.” Therefore the models were entered in the models gradually according to crescent (from minor to major) p-values, and later excluded from the model when they lost significance on the way. That is an automated strategy in the SPSS software. We extended the text in order to clarify this aspect.

*- The statistical analyses do not support all of the conclusions. In the "Author Summary" section, page 3, lines 11-12, the author claims that "Benznidazole (...) prevented the progression to cardiac, but not digestive CD forms." The current study cannot support the claim. The author can attest that the univariate Cox regression demonstrated a statistically significant association between the use of Benznidazole and the occurrence of cardiac disorder. Further studies would be necessary to attest to the efficacy of the drug as the manuscript currently described it.*

**Response:** *Benznidazole* use was protective for prevention both in univariate and in multivariable analyzes (and we also performed individual adjustments, with similar findings). Anyway, we toned down that text.

*Ethical perspectives*

*- The study does not presents concerns about ethical or regulatory requirements being met. It followed principles from the Helsinki declaration and was approved by their local Committee for Ethics in Human research. However, one of the attached documents presented by the authors reveals private information of participants, with their names and electronic record numbers. Recommendations to the authors are described in the "Summary and General Comments" section of this peer-review.*

**Response:** We erroneously uploaded the non-anonymized version of the database. We corrected this error. We also stated in the analysis that Helsinki’s declaration guidelines were strictly followed.

Reviewer #3: See attached review

--------------------

<b>Results</b></br></br>

-Does the analysis presented match the analysis plan?</br>

-Are the results clearly and completely presented?</br>

-Are the figures (Tables, Images) of sufficient quality for clarity?</br></br>

*Reviewer #1: - No detailed information on analyses plan/protocol for the study.*

*- Unclear how missing information on patients loss to follow-up were addressed for analyses. It would be useful to include sensitivity analyses.*

**Response:** Most variables analyzed as risk factors were collected at baseline and a rigid protocol for follow up. Therefore, and since the sample is not too big, there was no relevant missing data. Losses to follow up (51 patients who were not followed in 2019) were excluded from the study. We preferred to exclude these patients, as we could not guarantee that there was non-differential loss between exposures, which is an assumption of time-to-event models. The remaining differences in time of follow up were corrected by using Cox-regression models. In fact, they were not really “losses to follow up”, but rather patients who were more recently diagnosed, so that the follow up ranged from one (1998 through 1999) through 21 years (1998 through 2019).

*Reviewer #2: - The authors presented an exciting data set that can be appreciated by researchers from different research fields of neglected tropical diseases, including clinical and epidemiological insights that can help theorize some plans for pathophysiology investigations.*

*- The analysis presented matches the analysis plan. Surprisingly, the results on Benznidazole's use receive a higher focus than initially suggested by the "Introduction" or "Methods." The results on this anti-parasitic are indeed relevant. However, the authors should make that clear in the earlier sections of the manuscript, including its title, that they intend to give this much focus on the drug.*

*- The "Results" section revealed that of the 87 subjects who develop cardiac complications, and 100 subjects who develop digestive complications, 10 participants developed both. Nevertheless, the subjects who developed both complications are left are participants of the two primary outcomes. Perhaps the authors should consider if these 10 individuals should constitute a separate outcome group, as their susceptibility may differ. That way, there could be four outcome groups: 1- No change from ICF; 2- Cardiac complications; 3- Digestive complications; 4- Both cardiac and digestive complications. If this is taken under consideration, then table 1 should be modified. In consideration of four outcome groups, the 10 individuals should also be removed from tables 4 and 5.*

*Considerations for table 4 and their associated results:*

*- Should the 10 individuals who also presented digestive changes be accounted for inside this group?*

**Response:** We thank again Reviewer #2 for his/her kind comments. We changed introduction and title to increase the relevance of Benznidazole to our study. As for those subjects who developed both digestive and cardiac forms, we think the number is too small to warrant specific analysis. Therefore, we kindly ask the permission to maintain Table 1 in the present form. Of notice, even though all overlap cases were included in table 1, the Cox regression was performed only for the first outcome (for instance, if a patient developed cardiac form first, he was not included in the digestive form analysis; and vice-versa).

*- "Age" is statistically significant but is not all in bold, as suggested by the table's footnote. - "Hypertension" is statistically significant, but is not all in bold, as suggested by the table's footnote. There is no multivariable analysis demonstrated for "hypertension." - "Lung Disease" is statistically significant, but is not all in bold, as suggested by the table's footnote. There is no multivariable analysis demonstrated for "Lung Disease." There is no mention of "Lung Disease" in the "Results" section's text on page 6. - "Neurological Disease" is statistically significant, but is not all in bold, as suggested by the table's footnote. There is no multivariable analysis demonstrated for "Neurological Disease." There is no mention of "Neurological Disease" in the "Results" section's text on page 6. - "Tyroid Disease" is statistically significant, but is not all in bold, as suggested by the table's footnote. There is no multivariable analysis demonstrated for "Tyroid Disease." There is no mention of "Tyroid Disease" in the "Results" section's text on page 6. - "Charlson comorbidity index" is statistically significant, but is not all in bold, as suggested by the table's footnote. There is no multivariable analysis demonstrated for " Charlson comorbidity index." There is no mention of " Charlson comorbidity index " in the "Results" section's text on page 6*.

**Response:** All those errors were corrected.

- It is unclear if the authors only intended to perform multivariable analysis on the statistically significant variables from the univariate analysis. If that is the case, it is unclear why "hypertension","lung disease", "neurological disease", "tyroid disease" and "Charlson comorbidity index" had no multivariable analysis.- It is unclear what are the variables that are considered in the multivariable analysis.- It is unclear if confounders or effect modifiers were considered in the multivariable analyses, and if so, which ones.

**Response:** We tried to clarify the method for inclusion and exclusion of variables in the models, as follows, in the revised text: “Variables were included gradually in multivariable models using a stepwise forward strategy, i.e., according to a crescent order of p-values [17]. A final value of P<0.05 was used as criterium for entry permanence in the models. No variable was forced in the models. All data analyses were performed using SPSS 20 (IBM, Armonk, NY).” We hope the new text explains how we proceeded for including variables.

*Considerations for table 5 and their associated results:*

*- On page 6, lines 21-22, the authors say that "female gender was the only independent predictor of progression to digestive forms." However, table 5 reveals that "depression" was also an independent predictor. The authors fail to mention this relationship. - It is unclear if the author only intended to perform multivariable analysis on the statistically significant variables from the univariate analysis. If that is the case, it is unclear why "depression" had no multivariable analysis.- It is unclear what are the variables that are considered in the multivariable analysis.- It is unclear if confounders or effect modifiers were considered in the multivariable analyses, and if so, which ones.*

**Response:** After extensive discussion with fellow psychiatrists, we decided to remove the “depression” variable, as we found it was very subjective (though we at first considered based on the use of anti-depressants). A novel analysis presented similar results (indeed, the variable “depression” was excluded from the model based on our stepwise strategy, described in the previous response and in the revised text).

*Considerations for figure 1 and their associated results:- The survival functions described in figure 1 are related to the risk of developing cardiac complications (figure 1A) or digestive complications (figure 1B). The graph should be made clear, both in the title and the y-axis, to avoid confusion that the survival could be analyzing the risk of death.- It would be interesting to know how the results from table 1 and the association with Benznidazole behaves when accounting for other variables, such as "age," "hypertension," "myocardial infarction," and other pre-existing cardiovascular conditions.*

**Response:** We changed the figures to clarify meanings. We did assess the association with Benznidazole adjusted for heart diseases, age, hypertension and male gender. We chose to present the multivariable models only with the variables that were statistical significant in the last model, since we believe that this way of presenting results is more easily interpreted by the readers.

Reviewer #3: (No Response)

--------------------

<b>Conclusions</b></br></br>

-Are the conclusions supported by the data presented?</br>

-Are the limitations of analysis clearly described?</br>

-Do the authors discuss how these data can be helpful to advance our understanding of the topic under study?</br>

-Is public health relevance addressed?</br></br>

*Reviewer #1: Need to address/discuss in the conclusions quite a few issues and limitations: • Important to clarify the design of the study (if retrospective versus prospective)y• Standardisation of assessments or lack thereof• Ascertainment on treatment and low number of patients treated with Benznidazole• Lack of serological and/or parasitological response markers*

**Response:** We corrected all those aspects in the revised text.

*Reviewer #2: - The authors appropriately discuss the epidemiological importance of the study and how it particularly relates to Brazil, the country where the study has been conducted.- The "Discussion" section has a disproportional emphasis on statistically significant association observed between the use of Benznidazole and the progression to cardiac forms of Chagas' Disease. Unlike what the authors initially claim will be the primary intent of the study, the discussion and conclusion focus on the use of the drug and only make a brief mention of one of the possible predictors of disease: "hypertension."- The discussion and conclusion should be reworked to address the aims the manuscript initially sets. The last paragraph of the "discussion" section holds the claim that Benznidazole is beneficial for the prevention of progression of ICF to cardiac forms of Chagas Disease. However, this is not entirely supported by the data. The data only demonstrates a strong and statistically significant association between Benznidazole and the cardiac progression of Chagas' Disease. Although suggestive of a protective effect, not much was discussed on how other variables' presence may interfere with this association. The authors describe the limitation of the non-concurrent design of the study. Nevertheless, there could be a mention of other limitations, such as possible bias, confounders, and effect modifiers. The authors discuss how these data can help advance our understanding of the topic under study, but different than the initial aim of determining predictors of disease progression, it primarily focuses on Benznidazole's therapeutic potential.The authors present rich data and interesting results. Unfortunately, there are missed opportunities on result items that could or should have been addressed in the discussion. To name a few: The authors observe a statistically significant association between "age" and the development of cardiac problems. Despite the Chagas' disease diagnosis, age is usually associated with increased risk for cardiac complications. What hypothesis could be drawn? What could be discussed?The authors observe a statistically significant association between "hypertension" and the development of cardiac problems. Despite the Chagas' disease diagnosis, hypertension is usually associated with increased risk for cardiac complications, including cardiac failure, cardiac wall weakening, and cardiac hypertrophy. What hypothesis could be drawn? What could be discussed?The authors observe a statistically significant association between "myocardial infarction" and additional cardiac problems. Despite the Chagas' disease diagnosis, the occurrence of myocardial infarction is already associated with increased risk for cardiac complications, including cardiac failure, cardiac wall weakening, and cardiac hypertrophy. What hypothesis could be drawn? What could be discusseThe authors observe a statistically significant association between "lung disease" and the development of cardiac problems. Despite the Chagas' disease diagnosis, lung disease can have an independent relationship to the occurrence of right-sided cardiac complications. What hypothesis could be drawn? What could be discussed? The authors observe a statistically significant association between "neurological disease" and the development of cardiac problems. Why wasn't anything mentioned? What hypothesis could be drawn? What could be discussed? The authors observe a statistically significant association between "thyroid diseases" and the development of cardiac problems. Why wasn't anything mentioned? What hypothesis could be drawn? What could be discussed?The authors observe a statistically significant association between the "Charlson comorbidity index" and the development of cardiac problems. What hypothesis could be drawn? What could be discussed? The authors observe a statistically significant association between the "female gender" and the development of digestive problems. What hypothesis could be drawn? What could be discussed? The authors observe a statistically significant association between "depression" and the development of digestive problems. Female gender is usually associated with a higher incidence of depression. Would the accurate correlation remain with gender, or does depression play a role as a predictor? What hypothesis could be drawn? The authors did not observe a statistically significant association between "diverticular disease" and the development of digestive problems. Diverticular disease habitually happens due to the weakening of the intestinal wall. Why couldn't this have played a role in complications?*

**Response:** We totally agree with the reviewer that several important topics were missing in our discussion, so we did include more information. However, Reviewer #2 points to several associations which disappeared in the multivariable analyses (i.e., were probably confounded by the heart diseases or other concurrent aspects). Therefore we do not think it is appropriate to search for associations for lung diseases, thyroid diseases and the Charlson comorbidity index (which is a multivariate data that aggregrates comorbidities), which were probably confounded associations (correctly eliminated in multivariable analysis). As mentioned above, we removed depression from our analysis. For the remaining aspects pointed by Reviewer #2, we extended the discussion section.

Reviewer #3: (No Response)

--------------------

<b>Editorial and Data Presentation Modifications?</b></br><br/>

Use this section for editorial suggestions as well as relatively minor modifications of existing data that would enhance clarity. If the only modifications needed are minor and/or editorial, you may wish to recommend “Minor Revision” or “Accept”.

*Reviewer #1: There are a few typographic errors (Benzinadole; neglection; fibilation). Some conceptual issues: - though BENEFIT trial include long term patient follow-up, it did not include patients with chronic indeterminate Chagas disease, but patients with early cardiac disease manifestations); - difficult to state that almost all patients with Chagas live in South America (as stated in the introduction), when one has circa 40,000 patients in Spain and around 300,000 in the US.- parenteral transmission is not a recent form of spread of the disease (as stated in the Introduction), but rather it has been recognised for decades and control measures significantly reduced its impact)*

**Response:** We agree with Reviewer #1 and changed the text accordingly.

*Reviewer #2: - There is a disruption in the manuscript's flow. The "Title" and "Introduction" suggest that the manuscript's primary focus is to determine disease predictors for the development of cardiac and digestive disorders among patients with chronic indeterminate Chagas' disease. However, the center stage of the "Discussion" section is given to an elaborate review of the therapeutic potential of Benznidazole. The "Discussion" should be reworked, and the flow of the manuscript rearranged to reflect what the manuscript describes as its purpose. (Please see more details on the peer review feedback for "Results," "Conclusions" and ""Summary and General Comments").- Although some grammatical issues can be perceptible to a native speaker of American English, they are mild and do not interfere with the manuscript's comprehension.- On page 6, line 2, the drug Benznizadole has a typo "Benzinadole"*

**Response:** As aforementioned, we included the assessment of benznidazole among the objectives (including it even in the title). We changed the discussion in many aspects and performed extensive revision for syntax errors and typos.

Reviewer #3: (No Response)

*Reviewer #1: There is an important need for cohorts with long term follow-up of clinical outcomes in Chagas and the assessment of impact of different predictors of treatment response (including treatment). The study has some limitations which could certainly be addressed with significant revisions and clarifications. It is really unfortunate, however, that the supplementary information provided includes the name of patients and other identifying information. This represent a serious data privacy and ethical issue in implementation of clinical research, which I believe it would be challenging to resolve. This would require editorial consideration.*

**Response:** We deeply apologize for uploading the wrong database (we are uploading an anonymized database now).

*Reviewer #2: Summary*

*- The authors' interest in the topic of Chagas Disease and further discern its predictors is a commendable intention. As the authors also go to explain, despite the prevalence of Chagas Disease, especially its indeterminate chronic form, what is often offered to patients is not enough to improve prognosis and disease outcome. Therefore, the study's primary aim to better determine disease predictors is an objective of high relevance, both from research and clinical perspectives. Determining predictors for disease severity provides the opportunity to troubleshoot patient screening by focusing on particular characteristics.- However, there is a disconnect between what is described as the primary goal of the study (as described in its title, abstract, and introduction) and what the article focus on in its results, and more importantly, its discussion.- The introduction sets the stage for the importance of the article's primary aim: determining predictors for the development of cardiac and digestive disorders among patients with chronic indeterminate Chagas disease. Per this primary aim, the results describe the statistical significance of a set of characteristics that may play an important role as predictors of disease. However, the discussion falls short in delving into more details on the findings described in the results sections. Instead, it goes to focus on a hypothesized efficacy of the drug Benznidazole as a protective therapy for the cardiovascular outcomes of Chagas' disease.- Although there is a statistically significant association between the use of Benznidazole and the development of cardiac disorders among patients with chronic indeterminate Chagas Disease, this is a secondary aim of the study. More focus should be given to describing the results section's findings and establishing plausible hypotheses that justify all the statistically significant variables as possible predictors of disease severity. After all, this was said to be the goal of the study.- Furthermore, despite the Benznidazole's statistical significance, the findings are not sufficient to claim it as an efficacious drug. Additional studies are necessary to support this claim, including a study design that focuses on the outcomes of said drug and considers possible bias, confounders, and effect modifiers that were not fully explored in this study. As it stands, the use of Benznidazole has an association with the outcome, but causality cannot be suggested.- The study is relevant research that offers new data collected from patients, which can elicit essential discussions in the scientific community. However, when trying to focus on the drug, the authors miss the opportunity to engage in a fruitful discussion on the bulk of the obtained results.- The statistically significant variables that are possible predictors, and the not statistically significant variables that are discarded as predictors offer a relevant story that should be told, and hypotheses that should be discussed. I encourage the authors to rework the manuscript to establish a more explicit correlation between their aim of identifying predictors of disease outcomes, as they initially described in the "Introduction" and the details focused on the "Discussion" section.*

*Additional recommendations to the authors:- The attachment named "renamed_0bb9e.xlsx" contains patients' names and patient record numbers. I strongly suggest that the authors replace the document with a version that preserves the study participants' anonymity. Efforts should be taken to substitute patient identifiers with particular encrypted codes that only the research team could use to identify them. On the "Author Summary" section, page 3, lines 3-5; and "Introduction," page 4, lines 6-8, the idea of parenteral transmission through blood transfusion is described, also highlighting the risks of Chagas' disease transmission from the migration from South America to other parts of the world and the subsequent involvement of these individuals in blood transfusion. Although scientifically sound, the concept of the potential risk of South Americans' migration to other parts of the world should be treated with more responsibility in the manuscript. These particular sections of the manuscript could easily be taken out of context when presented to the public at large, reinforcing stereotypes and fueling xenophobia and racism again South American immigrants. The authors should make an effort in the passage's language to enforce the scientific interpretation and avoid any claims that could be taken out of context to propagate discrimination. I understand this was not the authors' intention, but I believe it is an important point to bring to the attention since the readers can be from areas facing different political climates.*

**Response:** The extensive comment underscores the careful reading of our manuscript, and we are grateful for that. So, we changed the text to include assessment of the use of Benznidazole as one of the main objectives of the study. We also performed extensive changes in the discussion.

Reviewer #3:

*The authors analyze data collected at their institution on 379 persons with the indeterminate form of Chagas disease followed during a 20-year period. Their study population is of special interest because of the high proportion of subjects with cardiovascular comorbidities and risk factors for cardiac disease (hypertension, myocardial infarction, hyperlipidemia, diabetes); in addition, the median age of subjects is substantially greater than that of earlier studies of younger cohorts of persons with the indeterminate form. As such, the authors' study population presents an opportunity to shed light on the evolution of Chagas heart disease among older persons and persons with comorbidities, who now represent a major proportion of persons infected with T. cruzi. However, as analyzed and presented, the study offers few insights into these important groups of persons with chronic infection, and conclusions about the impact of benznidazole on evolution of heart disease may be misleading. In contrast, the data and analysis on gastrointestinal disease seem more robust in part because few persons in the population had underlying gastrointestinal disease or neurological disease that might predispose to chronic Chagas GI disease. The authors do not define what is meant by the indeterminate form of Chagas disease. The traditional definition includes a normal electrocardiogram (ECG); however, a high percentage of participants had heart disease (defined in later tables as myocardial infarction) or hypertension, and I suspect that some (if not many of these) had some type of abnormality on ECG. Roughly 73% of persons who developed the cardiac form of Chagas disease had hypertension on initial examination and 32% had heart disease (“myocardial infarction”); the corresponding number of persons who did not progress were 50.5% (97/192) and 8% (16/192); respectively. The cardiac endpoints are defined as “abnormalities in those tests that could not be attributed to other causes” (table 2); RBBB is suggestive of Chagas disease, but neither RBBB or any of the listed abnormalities are completely specific for Chagas disease. LBBB (accounting for >25% of the abnormalities in table 2) occasionally can be attributed to Chagas disease, but is much more likely in this population to be a consequence of hypertension. The same can be true of LV dysfunction and other abnormalities in table 2. The methods section states that all studies were repeated yearly—if so, this would be an unusually high rate of follow-up for a large population followed over a 20 year period. However, some of the abnormalities listed in table 2, such as atrial fibrillation, dilated cardiomyopathy right, left bundle branch block and others would never develop in one year’s time in an infected person with a normal ECG and echocardiogram. In population-based studies of Chagas cardiomyopathy, the usual evolution is for early development of RBBB and then progression to more advanced disease; persons in the indeterminate stage who have not developed evidence of heart disease tend to remain in the indeterminate stage for decades. Many of the patients in the present study probably had been infected for many years or decades at time of entry, based on their ages and the fact that transmission of T cruzi had been interrupted in much of Brazil (and certainly in Sao Paulo state) prior to their enrollment. Thus, development of cardiac abnormalities may have been due to processes other than Chagas disease. If I’m reading the data in the supplementary file correctly, of the 56 persons with underlying heart disease, only 5 received benznidazole; the percentage of persons without underlying heart disease who received benznidazole is >2x greater; this suggests that presence or absence of heart disease may have influenced the decision to administer benznidazole. Given the absence of criteria that determined which patients received benznidazole, conclusions cannot be drawn about its efficacy in preventing the development of Chagas heart disease. In summary, the authors have compiled a potentially important data set that could shed light on the evolution and prognostic factors for Chagas heart disease in persons with comorbidities, cardiac risk factors, and older age. The authors should consider reanalysis of the data comparing outcomes in persons with pre-existing conditions to those without and controlling for age. Given the small numbers of persons who received benznidazole, it may not be possible to draw conclusions about the effect of benznidazole in different groups. As the authors acknowledge, randomized controlled studies are essential for evaluating the impact of benznidazole. Specific minor comments: Page 2, lines 6-7. The authors might point out that the study was carried out at a time in which most transmission in Southeastern Brazil had been interrupted.Page 3. Migration is unlikely to lead to major spread of infection within Europe in North America by transfusion or other routes Page 4. Line 3. Many persons with Trypanosoma cruzi infection live outside of South America; would not say that almost all of them live in South America. Line 5. Oral and parenteral transmission has been known for a long time; would not say that they were reported more recently. Line 7-8. Extensive migration has already led to large numbers of infected persons in Europe North America. Page 5. It would be important to clearly define what the authors mean by indeterminant form of Chagas disease. As mentioned above, many of the subjects had a history of underlying heart disease and may not fit the strict definition of indeterminant form.Table 1. "Heart disease" later is defined as myocardial infarction. Table 3. Would define what is meant by “colon abnormalities”Figure 1. The curve extends to greater than 400 months although the study lasted only 240 months. Not clear which meant by cumulative survivalPage 15. Line 2-4. It was not just urbanization and improvement of housing but also the national control program that reduced transmission of the parasite. Line 6. If there are 7 million infected persons, greater than 1-2 million would have the indeterminate form. Line 11-12. Rate of progression from indeterminate form to cardiac form varies by age and is not 5% across all age groups.*

**Response:** Since Reviewer #3 sent hers/his comments as an attached document, many of the points made in the comments were already addressed above (I kindly refer Reviewer #3 to previous response). We will address other aspects individually here. In order to make clear some of the aspects posed by the reviewer we: *(i) included in the text a definition of indeterminate, as a patient not presenting cardiac or digestive abnormalities* ***attributable to Chagas Disease*** *in initial assessment; (ii) coherently, we included a table (new table 1) presenting our protocol for initial assessment and follow-up of patients, in terms of clinical assessment and performing ECG and imaging diagnoses*. *Even though RBBB is not pathognomonic of CD, the development of this disturbance in a patient with previous normal ECG was assumed to be a diagnostic criteria for cardiac form.* The new analyses recommended by the Reviewer may be of interest, but we think that over-stratification of a relatively small sample will be unfruitful. Anyway, the rationale of data availability is to allow other researchers to re-analyze our data, possibly providing novel insights. We do agree that many infected persons already live in Europe and US, therefore we made a little change in our text to make clear that this, in our opinion, poses a risk for transfusion and vertical transmission. We hope the revised text is more in accordance with Reviewer #3’s requirements.

Finally, and having performed peer-reviews ourselves, we consider that this process is a “conversation between intelligent people” (to borrow a quote by Ezra Pound referring to literary criticism). We are greatly indebted to the reviewers for a rigorous assessment of our study and for recommendations that undoubtedly improved our text.

Yours,

Erika Alessandra Pellison Nunes da Costa

Cassiano Victória

Carlos Magno Castelo Branco Fortaleza
