# Supplementary material for: Predictors of development of cardiac and digestive disorders among patients with indeterminate chronic Chagas Disease
Source: PLoS Negl Trop Dis. 2021 Aug 13;15(8):e0009680. doi: 10.1371/journal.pntd.0009680 (PMC8386853; doi:10.1371/journal.pntd.0009680)
Supplement: Attachment — Submitted filename: Response to Reviewers #2.docx [file pntd.0009680.s005.docx]

RESPONSE TO REVIEWERS

To the Editor and Reviewers –

The authors thank you for the revision and recommendations to our manuscript. We tried our best to follow the suggestions and changed the text accordingly. The specific topics are addressed bellow:

# Reviewer #2

*The objectives of the study were clearly articulated with a clear testable hypothesis. The clarity of the aims has improved since the original submission. This time, two aims were characterized. The first is to assess possible variables/predictors that contribute to the evolution of the indeterminate chronic form of Chagas disease into the disease's cardiac or digestive conditions. The second, to assess the impact that Benznidazole has as a therapeutic intervention in ICF of Chagas disease, focusing on its ability to prevent the progression to cardiac and/or digestive forms of Chagas disease. The study design appropriately addresses the stated objectives. A non-concurrent cohort study analyzing data collected from Chagas disease patients being followed prospectively in the outpatient clinic over two decades. The study's data was then retrieved retrospectively from their medical charts. The population was clearly described and seemed appropriate for the hypothesis being tested. The study group recruited Chagas' disease patients from the Tropical Diseases outpatient service in Botucatu Medical School, São Paulo State University (UNESP), who presented with the indeterminate chronic form of Chagas disease from the year 1998 through 2018, with an extended follow-up period to December 2019. The inclusion criteria demanded a Chagas disease diagnosis based on positivity in two serological tests (ELISA and indirect immunofluorescence). Only patients who were positive in both were included in the study. As exclusion criteria, those who presented abnormalities that could be attributed to either cardiac or digestive forms of CD upon admission were excluded from the study, which was an appropriate measure since the group wanted to assess the risk of evolving in these conditions and not recruiting patients who already had them. Patients lost to follow-up were also excluded from the study. A noteworthy mention is that this data comes from a specific city of Brazil with a particular set of demographic characteristics. Therefore****, caution should be taken when discussing the data's generalizability to other populations who don't share all features.*** *However, the study's publication will be interesting to other groups who could then attempt to reproduce the design in different regions of Brazil or country, further assessing similarities and differences in the patterns gathered from other populations.*

**Authors’ response:** We thank Reviewer #2 for her/his extensive analysis of our study. We revised the text avoiding inappropriate gerenalization.

***No mention is made in the manuscript to describe if the sample size is sufficient and ensure adequate power to address the tested hypothesis. However, the study uses a convenient sample of all the patients with a Chagas Disease diagnosis admitted while presenting the disease's indeterminate chronic form. The study does yield statistical significance.***

**Authors’ response:** Even though we used a convenient sample of all outpatients meeting inclusion criteria, we performed a post hoc analysis of study power using OpenEpi software (Emory University, Atlanta, GA). We estimated the study power based on the impact of Benznidazole use and found it to be 89.7% and 96.1% for association with progression to cardiac and gastrointestinal CD.

*Statistical analyses utilized univariate and multivariable Cox regression models, which could be considered appropriate to assess associations between particular variables and outcomes of interest as set by the study's two primary goals. There are no current concerns about ethical or regulatory requirements. The study was exempt by the local IRB, and approval information was provided. Also, the breach of patient confidentiality exhibited in the original submission, which contained patients' names and chart numbers, were addressed in the authors' responses to reviewer comments and fixed in the new submission.*

**Authors’ response:** We thank Reviewer #2 for her/his comments.

*- The analysis presented matches the analysis plan. - Tables 1 and 2: Classically, subjects characteristics demographic information is shown in table 1. In other words****, invert the order of the current tables 1 and 2****. - Table 3: please see suggestions in the "Editorial and Data Presentation Modifications" section. Table 4: no issues. - Tables 5 and 6: In the multivariable analysis column, are the lines in blank not tested or just not significant. If tested, please include them in the table. - Emphasis was given to portraying the results through tables and figures. However, having some highlights in the results could help the reader be more familiar with the results that the authors considered to be most relevant.*

**Authors’ response:** We kindly ask Reviewer #2 to allow the current order or tables, since **Table 1** refers to methods and **Table 2** to results. In the multivariable analysis of **Tables 5** and **6**, the lines in blank represent variables not included in the final step of stepwise multivariable analysis (regardless of their significance). We included that information in the note bellow the table. Finally, we included some highlights of the results in the manuscript text.

*- The discussion section has a statement* ***"Data from literature agree with our findings," which findings are the ones referred to by the authors?*** *- The first part of the discussion focuses on mentioning other studies but fails to appropriately discuss the results found in table 4, table 5, table 6, figure 1, and figure 2. - The discussion only briefly introduces the discussion on the cardiovascular form of Chagas disease but only focuses on the systemic arterial hypertension component of the results. The discussion should expand on the interpretation of the cardiovascular findings from the results section.- The discussion doesn't delve into the digestive form of the disease, which was one of the study objectives. It only mentioned the digestive complication concerning the use of Benznidazole. -The authors discuss how these data can be helpful to advance our understanding of Chagas disease. - The public health relevance of the manuscript is discussed in the abstract and conclusion.*

**Authors’ response:** The findings referred in the discussion are those regarding the impact of age and comorbidities. We clarified this in the discussion. We also extended the discussion to discuss interpretation of our findings for both outcomes of interest.

*A minor suggestion is the change of table order. Classically, subjects’ characteristics and demographic information are shown as the “table 1”. In other words, I suggest inverting the order of the current tables 1 and 2. 2- I understand the attempt of blacking out the cells of table 1 to make it more visually appealing, but I believe it is not entirely intuitive for all readers. I would suggest reworking the visual aspect of the current table 1. Some suggestions would be having two columns, the second column explaining when the assessment is done. Another option is to substitute the black and whites with “Yes” and “No.” 3- I appreciate that the title now has a mention of Benznidazole therapy. Since the Benznidazole therapy is a second aim and not a focus of the first aim, I would suggest that rather than saying “emphasis on,” the authors could adopt “and.” The authors also call the study disease ICF “indetermined chronic form,” but in the title, they display a different order of the words like “chronic indeterminate Chagas.” I would then suggest adapting the disease name in the title to match the rest of the manuscript. The proposed title would then read: “Predictors of development of cardiac and digestive disorders among the indeterminate chronic form of Chagas Disease and the impact of Benznidazole therapy.” 4- A few times in the article, the disease of interest is described as “indetermined chronic form (ICF)”; other times, it is called “chronic indeterminate,” including in the tables. I suggest using only one form to keep consistency. 4- Most of the work that needs addressing is in the discussion section. The study's primary aims are to identify predictors that would influence the progression of Chagas disease in its indeterminate chronic form to either the cardiac form of the disease or digestive condition and the impact of Benznidazole therapy as a possible protector. The discussion spends its first few paragraphs discussing other publications. Then, it only highlights one of the results pertaining to the cardiac form of the disease and only discusses the digestive form related to the use of Benznidazole. The discussion should be used as an opportunity to highlight the results and how they address the study’s objectives, including the association with possible predictors for the cardiac and digestive forms of the disease, and then if Benznidazole had an impact and if that was protective.*

**Authors’ response:** Regarding **Tables 1** and **2**, please see response to previous comments. Regarding the reference to Benznidazole in the title, we seem to have a disagreement among reviewers (in fact, Reviewer #3 recommends not focusing tile, analysis and discussion on the therapy). This is rather common and is in line with Karl Popper’s view that science progresses through an “educated network of mutual criticisms” (Popper K. Conjectures and refutations. London: Routledge, 2020). We emphasize that: (i) investigating the impact of Benznidazole was a secondary objective in our study; (ii) the cohort study (especially a nonconcurrent cohort) is not the most appropriate analyses for a therapeutic approach. Therefore, we attempted to change the title to leave it halfway between Reviewer #2 and Reviewer #3 recommendations. We also toned down some conclusions regarding the impact of Benznidazole. The discussion section was thoroughly revised.

*6- Other minor changes: 5.1- Doença de Chagas can be represented as “Chagas Disease,” without the need for an apostrophe to designate possession of a noun ended with “s.” The manuscript has a recurrent variation as “Chagas’ Disease,” which could be modified to “Chagas disease.” 5.2- Table 2 note says, “Statically,” please modify to “Statistically.” 4.3- Table 3 designates ventricular disfunction as “DV.” I suggest adapting to “VD” to match the other acronyms in the table. 5.4- The table has a typo “Dilateed,” please fix it to “Dilated.” 5.5- Please review the manuscript for other small typos or words kept in Portuguese.*

**Authors’ response:** We extensively revised the text to remove typos.

*The study sets to determine predictors for the development of cardiac and digestive disorders among patients with chronic and indeterminate Chagas Disease. The study's purpose is outstanding. Such determinants can enlighten clinicians on the particular variables that are most relevant for monitoring patients and ascertain the medical team's conduct. The study also wants to assess Benznidazole therapy outcomes and if such drug is associated with a protective effect to prevent the development of the cardiac or digestive form of the disease. The findings would also have the potential to establish a new hypothesis to incentivize new research avenues to study Chagas Disease. In this revised manuscript, the authors addressed most of the reviewers' concerns with the original submission. This research is worthy of publication, but the authors are having some issues in conveying the importance of their findings. Interesting data is presented in the results section, but not much of it is being portrayed or highlighted in the discussion section. Most of the manuscript only requires some fine adjustments. However, the discussion and conclusion are what most need to work. Most of the information is relayed with tables and figures rather than the text in the results section. This is an understandable strategy, which would also avoid the redundancy of having the same thing stated in text and tables. However, this leaves the discussion as a stage where the authors should highlight their findings and what they mean. However, the initial part of the discussion focuses on reiterating information from other publications. The middle part of the discussion mentions systemic arterial hypertension as a highlight of the cardiac form of Chagas disease but makes no further mention of other predictors. And the only comment on the digestive form of the disease is to outcomes related to the use of Benznidazole.The authors are on the right track, and their work is worthy of publication. However, they need to tie the discussion and conclusion back to the goals they described in the introduction and methods. With that said, please make sure the discussion section is highlighting the findings pertaining to predictors of the cardiac form of Chagas disease, highlighting predictors of the digestive form, and only then discusses the findings as they relate to Benznidazole. If the authors address the minor concerns and make sure the discussion correlates their findings to their aims, I believe the manuscript will likely be ready for publication.*

**Authors’ response:** The discussion was revised according to the reviewer’s recommendations.

# Reviewer #3

*The authors have addressed many of the comments raised by the reviewers of the original manuscript. The objectives are clearly stated and address critical questions about Chagas disease, but there are flaws in the study design, particularly pertaining to heart disease. The analysis for gastrointestinal disease is more robust. (1)* ***The authors do not define "abnormalities that could be attributed to chronic Chagas disease" when defining indeterminate Chagas disease; given the high percentage of persons with important cardiovascular comorbidities, there is a real possibility of misclassification. The authors should clearly elucidate which abnormalities were permissible for inclusion in their cohort. The classic definition of indeterminate Chagas disease is normal history, physical exam, chest xray and ECG; the authors could consider a new definition that includes persons with comorbidities.*** *(2)* ***The criteria for selecting which persons received benznidazole are not spelled out. Analysis of persons with and without certain comorbidities suggest bias that favors the effectiveness of benznidazole therapy. For example, Only 9% persons with pre-existing cardiac disease received benznidazole as opposed to almost 20% of those classified as not having cardiac disease upon entry. Similarly, only 15% of those with hypertension received benznidazole as opposed to 22% who did not have hypertension. 4. The authors do not define the timing of benznidazole therapy. Periods of follow-up after administration of benznidazole may be shorter than the periods of follow-up of persons who did not receive benznidazole.***

**Authors’ response:** We thank Reviewer #3 for the thorough methodological analysis. We did include subjects who presented heart diseases that could be attributed to coronary syndrome, myocardial infarction or hypertension and without typical findings of cardiac CD (dilated myocardiopathy, atrioventricular blocks, branch blocks). We agree that including those patients could lead to confounding. However, the Cox-regression analysis presented in the results adjusted the impact of Benznidazole for that variable. Of note, when we perform stratified analysis, we find that among 264 patients without “heart diseases”, 64 received Benznidazole, of whom 4 (6.3%) developed typical cardiac CD; 259 did not receive, and 55 (21.3%) developed cardiac CD. The risk ratio of Benznidazole use among persons without “heart diseases” was 0.29 (95%CI, 0.11-0.78) and Chi-square p-value of 0.003. Among 56 patients with heart disease, 5 received Benznidazole (2 developed cardiac CD) and 51 did not receive (26 developed cardiac CD). In that group, RR=0.78 (95%CI, 0.26-2.37). That means that the impact of Benznidazole was even greater among those who did not present heart diseases. A similar picture was found hypertension, with significant negative association of Benznidazole and progression for cardiac CD for those who did not have hypertension (p=0.02) and a only marginal significance (p=0.08) among those who did. Therefore, even considering confounding, it could underestimate (not super-estimate) the impact of Benznidazole in preventing cardiac CD. Doctors attending indeterminate CD patients used Benznidazole soon after diagnosis and according to the Brazilian Ministry Protocol, which is similar to US Centers for Disease Control and Prevention recommendations. Benznidazole was administered for two months. We changed the manuscript text in order to clarify issues pointed by the Reviewers. Finally, we included in the discussion the alternative Cox regression analysis of progression to Cardiac CD, removing from the study all those patients with either heart diseases or hypertension. The adjusted HR was 0.18 (95%CI, 0.06-0.50; *P*=0.001). The Figure bellow (Kaplan Meier graphic) was added in the discussion, in order to clarify that issue. Notice that the log-rank P-value was 0.001.

***4. There is likely to be misclassification of outcomes. For example, isolated BASRE (in the spread sheet) is defined as a specific endpoint for Chagas disease. (This is much less specific than right bundle branch block, but has high specificity when seen with RBBB.) It is well known that isolated BASRE (also known as left anterior hemiblock or anterior fascicular block) is common among person with hypertension and its prevalence increases with age. In the Excel sheet there are 21 persons who developed BASRE; 13 had preexisting hypertension (62%); only 8 persons of 163 without preexisting hypertension developed BASRE (5%); all 3 persons who had complete left bundle branch had preexisting hypertension.(note that BASRE + LBBB =the 24 persons with LBBB in table 3).Moreover, the persons who developed BASRE are older at endpoint (average 67.9 years) than those who did not develop BASRE (61.9 years). Note--would not lump LBBB and LAH into one category.***

**Authors’ response:** We admit that misclassification bias can occur. We attempted to avoid confusion by adjusting pre-existing disorders and endpoints, whenever we had enough statistical power. We included misclassification bias as a possibility among the study limitations. Even with that limitation, our findings point out to a beneficial impact of Benznidazole.

*Presentation is clear. The discussion and conclusion seem to focus on Benznidazole and miss the opportunity to discuss the results on the predictors for the cardiovascular and digestive forms of Chagas Disease. Several conclusions, especially regarding benznidazole therapy, are not supported by the data presented for reasons above. The authors nicely address other aspects of the study in their conclusions.*

**Authors’ response:** We revised the discussion extensively to tone down the aspects regarding Benznidazole therapy.

***Would suggest reanalysis of this rich data set to look at the importance of comorbidities on the evolution of chronic Chagas heart disease as well as characteristics of Chagas disease in older populations****. The raw data themselves are important! A purely descriptive paper about evolution of Chagas disease in older persons and persons with comorbidities would be a terrific contribution to the literature. Would deemphasize data on benznidazole, which are misleading. The analysis of gastrointestinal disease is sounder.*

**Authors’ response:** As previously mentioned, we toned down the discussion regarding therapy, and focused on other risk factors, an aspect in which both reviewers agree. However, as we also discussed above, Reviewer #2 praises the authors for including Benznidazole in the title (more emphasis on Benznidazole was a recommendation in the previous review). We repeat here our comments to Reviewer #2: Opposite recommendations by reviewers are rather common and are in line with Karl Popper’s view that science progresses through an “educated network of mutual criticisms” (Popper K. Conjectures and refutations. London: Routledge, 2020). We emphasize that: (i) investigating the impact of Benznidazole was a secondary objective in our study; (ii) the cohort study (especially a nonconcurrent cohort) is not the most appropriate analyses for a therapeutic approach. Therefore, we attempted to change the title to leave it halfway between Reviewer #2 and Reviewer #3 recommendations.

*The strength of the paper is the study population: persons with indeterminate Chagas disease, many of who have important comorbidities and are older than subjects of earlier studies of the natural history of Chagas disease. The study has the potential to fill important gaps in our understanding of chronic Chagas disease. Would suggest reanalysis of data looking at outcomes as a function of comorbidities and analyze data without (as well as with) benznidazole treatment.*

**Authors’ response:** We perform reanalysis removing Benznidazole as a variable and included a brief comment in the discussion section. Even though including analysis in the discussion is not a usual approach, we believe that it emphasizes our line of thought and provides a “counterfactual” approach to our inferences.

# FINAL COMMENTS

Our study aimed at contributing to understanding natural history of Chagas Disease, as well as to discuss (not conclusively) the role of antiparasitic therapy. While many limitations remain (and they are emphasized in this newly revised version), we stand for the scientific value and practical relevance of our findings. The reviewers’ recommendations certainly improved our study inferences, and therefore we are indebted to their insights.

Yours,

Erika Alessandra Pellison Nunes da Costa

Cassiano Victória

Carlos Magno Castelo Branco Fortaleza
