# Supplementary material for: Predictors of development of cardiac and digestive disorders among patients with indeterminate chronic Chagas Disease
Source: PLoS Negl Trop Dis. 2021 Aug 13;15(8):e0009680. doi: 10.1371/journal.pntd.0009680 (PMC8386853; doi:10.1371/journal.pntd.0009680)
Supplement: Attachment — Submitted filename: RESPONSE TO REVIEWERS #3.docx [file pntd.0009680.s006.docx]

RESPONSE TO REVIEWERS #3

To the Editor and Reviewers’ –

We thank you once again for the time and effort dedicated to extensive review and recommendations to our study. Throughout the review process, we attempted to improve our manuscript to meet reviewers’ requirements. It has been a profitable dialogue, which is continued bellow.

**EDITOR’S COMMENTS**

*(…) Although the manuscript is improved, there are still some problematic concerns about the potential of misclassification bias regarding cardiac disease in the design of the assessment of benznidazole treatment. Please see the comments from reviewer 3. The analysis of impact of benznidazole on progression of heart disease is severely flawed, and confounding factors have not been sufficiently addressed. If possible, better adjustment for these factors would help, otherwise they must be discussed as flaws in the study design and the findings of the cardiac patient analyses reconsidered in that light.*

**Authors’ response:** We partially agree with the flaws. However, as we mentioned in our previous response to reviewers (specifically for Reviewer #3), a separate analysis excluding the two cited confounders (heart disease and hypertension, see bellow) presented similar results regarding the use of benznidazole. Furthermore, other reviewers welcomed our emphasis on benznidazole. We toned down the emphasis on benznidazole, removing it from the title. As for outcome misclassification, we included this possibility as a study flaw.

**REVIEWER #2**

*The following points deserve clarification in the Methods section: 1-	Define Benznidazole criteria if available: The authors mention that “Benznidazole was used when indicated by the attendant doctor, based on the Brazilian Ministry of Health recommendations,” however, other than pointing the reader towards the reference, the clinical criteria for these patients’ eligibility for Benznidazole was not defined. Please outline the characteristics used by the team to define eligibility. Since the Benznidazole therapy was studied as a variable of preventive association with the onset of cardiac and digestive forms of Chagas, it is important to understand why certain patients may have been eligible for the drug while others were not under the Brazilian guidelines. Not all patients are eligible for antiparasitic treatment, and the reasoning for prescription or contraindication may also be due to characteristics that may interfere with the statistical analysis or serve as confounders. However, considering the retrospective nature of the study and that data were from charts, it is possible that the authors did not have access to the physicians’ reasoning and criteria, just if Benznidazole had been used or not. If authors had no access to the criteria utilized by the attending physicians, this should be specified in the methods.2-	Better characterize which parameters were used for exclusion criteria: “Those who presented abnormalities that could be attributed to either cardiac or digestive forms of CD upon admission were excluded from our study.”, “our inclusion criterium was the admission with ICF, defined as “serological positivity for CD, without presenting any cardiac or digestive abnormalities that could be attributed to that disease.” Please characterize which parameters were considered for the exclusion of participants. Was it based solely on ECG cardiological findings like outcomes in table 3, or did it consider chest radiography, echocardiogram like table 1? For example, for the cardiologic form of CD: “dilated cardiomyopathy, congestive heart failure, arrhythmias, cardioembolism, thromboembolic events, stroke….”3-	Define “heart disease”: A point brought up in previous submissions and not yet fully addressed is what is being considered “heart disease.” In some tables, this is not specified. In others, it is suggested to be categorized as coronary diseases “Heart diseases not attributed to Chagas Disease were restricted to coronary syndrome, with or without myocardial infarction.” Please clarify the author’s definition of “heart diseases” in the method section. The conclusions have been made more evident in this submission. In addition, the authors have expanded the discussion to other predictors rather than the extended focus on Benznidazole therapy that had been given in previous submissions. Most limitations were acknowledged, and additional quantifications were introduced to address some of these imitations. The authors define the criteria for outcomes in the "Results" section. These are often described in "Methods," the switch could contribute to clarity.- The manuscript contains minor typographic errors throughout the text. For example, some words are in Portuguese, such as "áreas" in the tables, ICF is once referred to as IFC, missing spaces, missing periods, etc. Please review the entire manuscript for typos.*

**Authors’ response:** We do agree that criteria for administering benznidazole could be confounders of progression to localized forms. Still, we emphasize that we adjusted our analysis for those results in multivariable Cox models. We included this in the limitations section, which was extended. Exclusion was based on chest R-ray, digestive imaging, echocardiogram and ECG, as stated in **Table 1**. We clarified this item in the manuscript. We kindly ask the reviewer to consider the structure we used for the article sections, since we believe that it contributes to clarity. We extensively copyedited the manuscript.

**REVIEWER #3**

*Objectives are clearly stated and overall design appropriate. Problem with study is emphasis on evaluation of impact of benznidazole therapy, which is compromised by confounding. For example, just looking at the variable "heart disease", benznidazole was given to a lower proportion of persons who had preexisting heart disease on entry to the study (5/56) than those who didn't have heart disease (64/323); 28/56 of persons with preexisting heart disease had progression of heart disease; only 59/323 persons without preexisting heart disease developed heart disease. A quick look at table 2 shows a number of other comorbidities that predispose to heart disease (hypertension, diabetes) that are disproportionately distributed between the 2 outcomes and are also likely sources of confounding. Another major problem is misclassification of outcome (table 3): in this study LBBB is a more frequent outcome than RBBB; while LBBB is common in persons with other forms of heart disease (hypertension, LVH, aortic stenosis, etc) it is seen in a minority of persons with Chagas disease as the only cause of heart disease. Statistical analysis appropriate; no ethical concerns. (…) 2.Results are clearly presented but not completely presented. Results of simple analysis showing treatment status of persons with pre-existing conditions and those without would allow the reader assess possible confounding. The conclusions have been made more evident in this submission. In addition, the authors have expanded the discussion to other predictors rather than the extended focus on Benznidazole therapy that had been given in previous submissions. Most limitations were acknowledged, and additional quantifications were introduced to address some of these imitations. The authors discuss how these data can be helpful to advance our understanding of indeterminate chronic Forman acknowledge that proper clinical trials are necessary to address the suggested benefit from Benznidazole.Conclusions about impact of comorbidities on progression of heart disease are sound; conclusions about impact of benznidazole therapy are not. Conclusions about impact of benznidazole on progression of gastrointestinal disease are on more solid grounds because of less potential confounding and more precise definitions of outcomes. Rather than defining their population as persons in the indeterminate stage of Chagas disease, the authors should distinguish between persons without evidence of heart disease and persons with evidence of heart disease other than Chagas disease. The authors do not discuss potential confounding and misclassification. Discussion of importance of comorbidities on progression of Chagas disease is appropriate. Conclusion that controlled trials are needed is sound.* *Strengths of the study are the unique patient population of persons with T cruzi infection--they are older and have a high rate of well defined cardiovascular comorbidities and risk factors than persons in many other studies of Chagas disease. I believe that this is the great strength of the data set and the paper, and the authors' discussion of this is a strength. Weakness of the study is the emphasis on impact of benznidazole therapy on outcome of cardiovascular disease. Would recommend not making this an objective of the study; ok to present the data and observations but would make the point that conclusions cannot be drawn because of potential confounding; would also make clear that case definition (of indeterminate form that includes cardiac conditions not felt to be from Chagas disease) is not conventional, and that certain cardiac outcomes (eg, LBBB) are more typical of other heart diseases than Chagas heart disease. The analysis of impact of benznidazole treatment on gastrointestinal outcome is more robust because there is less potential confounding and the outcomes are more precise.*

**Authors’ response:** We addressed most of this aspects above in the response to the editor. As we mentioned above, we toned down the relevance of benznidazole (and changed the title), without completely excluding or changing our results. We did extend our limitation section, admitting flaws that we could not avoid. We believe that eve with these flaws the study contributes to the understanding of the evolution of Chagas’s disease. We kept the term “indeterminate form” because it is classic, even though we are aware that, using ultra-sensitive tests, minor abnormalities may be found in those patients. Finally, we thank once again the reviewers for their always rigorous (and at the same time kind) evaluation of our study.
